# Datasets for calcium dynamics comparison between the whole-cell and a β-escin based perforated patch configuration in brain slices from adult mice

**DOI:** 10.1016/j.dib.2021.107494

**Published:** 2021-10-17

**Authors:** Simon Hess, Christophe Pouzat, Peter Kloppenburg

**Affiliations:** a Biocenter, and Cologne Excellence Cluster in Aging Associated Diseases (CECAD), Institute for Zoology, University of Cologne, Cologne, Germany; b IRMA, Strasbourg University and CNRS UMR 7501, Strasbourg, France

**Keywords:** Calcium imaging, Added buffer approach, Perforated patch clamp, Dopamine neuron, Substantia nigra, Beta-escin, Calcium dynamics

## Abstract

Multiple processes shape calcium signals in neurons. The spatial and temporal dynamics of these signals are determined by various cellular parameters, including the calcium influx, calcium buffering, and calcium extrusion. The different Ca^2+^ handling properties can be estimated using the ‘added buffer approach’ [1], which is based on a single compartment model of Ca^2+^ buffering. To use this approach, the cell has to be loaded with a Ca^2+^ sensitive dye (e.g., fura-2) via the patch pipette, which is usually done in the whole-cell patch clamp configuration. However, determining Ca^2+^ handling properties can be complex and frequently unsuccessful due to the wash-out of intracellular components (e.g., mobile Ca^2+^ buffers) during whole-cell patch clamp recordings. We present two Ca^2+^ imaging datasets from adult substantia nigra dopamine neurons where the 'added buffer approach' was either combined with the 'conventional' whole-cell configuration or with a β-escin based perforated patch clamp configuration. These data can be used to compare the two methods or to draw comparisons with the Ca^2+^ handling properties of other neuron types. Further details and an in-depth analysis of the new combination of the ‘added buffer approach’ with the β-escin based perforated patch clamp configuration can be found in our companion manuscripts “Analysis of neuronal Ca^2+^ handling properties by combining perforated patch clamp recordings and the added buffer approach” [2] and “A Simple Method for Getting Standard Error on the Ratiometric Calcium Estimator” [3].

**Specifications Table**

**Table:** 

Subject	Neuroscience: Neurophysiology
Specific subject area	Cellular calcium dynamics. Substantia nigra dopaminergic neurons.
Type of data	Raw data: output of the CCD chip used to measure calcium-induced changes in fura-2 absorption.
How data were acquired	Fluorescence measurements were performed with fura-2 using an Imago/SensiCam CCD camera with a 640 × 480 chip (Till Photonics, Gräfelfing, Germany) and a Polychromator IV (Till Photonics). Both were mounted on an upright microscope. The Substantia nigra dopaminergic neurons were recorded in brain slices of adult mice.
Data format	HDF5
Parameters for data collection	Regularly timed excitations at three different wavelengths allowed the calcium dynamics to be monitored. The data are collections of time series recorded at 340, 360, and 380 nm excitation.
Description of data collection	Two different protocols were used for loading the calcium dye (fura-2) into the neurons: the ‘conventional’ whole-cell and the β-escin based perforated patch configuration.
Data source location	University of Cologne, Institute for Zoology Cologne, NRW Germany
Data accessibility	Repository name: Zenodo Data identification number: 4326607 Direct URL to data: https://zenodo.org/record/4326607
Related research article	S. Hess, C. Pouzat, L. Paeger, A. Pippow, P. Kloppenburg, Analysis of neuronal Ca^2+^ handling properties by combining perforated patch clamp recordings and the added buffer approach, Cell Calcium, 2021 May 10;97:102411. https://doi.org/10.1016/j.ceca.2021.102411 [3]

**Value of the Data**

• The data demonstrate the gain resulting from the proposed β-escin based perforated patch method when investigating calcium dynamics. They constitute a reference against which calcium dynamics studied in other cell types can be compared.
• The data are interesting for scientists working on neuronal (or more generally cellular) calcium dynamics and scientists developing analysis methods for fluorescence indicator-based calcium concentration studies.
• The data can be used for investigating the pros and cons of several acquisition protocols in fura-2 calcium measurement, e.g., the excitation wavelengths to use (three wavelengths were used here) or the sampling frequency. They can also be used for experiment planning: how long will a complete set of measurements require with the β-escin perforated patch method, how many transients can or should be used?
• The data allow anyone to reproduce the analysis presented in the companion paper “Analysis of neuronal Ca^2+^ handling properties by combining perforated patch clamp recordings and the added buffer approach” in combination with the publicly available analysis software (https://gitlab.com/c_pouzat/beta-escin-analysis).

## 1 Data Description

Our data are stored in two directories containing β-escin perforated patch recordings (‘data_beta_escin’) and whole-cell recordings (‘data_whole_cell’) of substantia nigra dopamine neurons. The HDF5 file format (www.hdf5.com) is used throughout. There is one file per experiment. Our file names DA_YMD_EN.h5 have the following meaning: ‘DA’ stands for dopaminergic neuron; ‘Y’ is the year (after 2000); ‘M’ is the month; ‘D’ is the day; ‘E’ stands for experiment and ‘N’ is the experiment number at that date.

Data_whole_cell contains data from 8 experiments:

DA_120906_E1.h5
DA_121011_E2.h5
DA_121015_E1.h5
DA_121108_E1.h5
DA_120913_E7.h5
DA_121011_E3.h5
DA_121015_E3.h5
DA_121108_E3.h5

Data_beta_escin contains data from 16 experiments:

DA_121219_E1.h5
DA_130130_E2.h5
DA_130514_E5.h5
DA_130531_E1.h5
DA_121219_E7.h5
DA_130130_E4.h5
DA_130523_E1.h5
DA_130531_E4.h5
DA_130128_E1.h5
DA_130201_E2.h5
DA_130524_E4.h5
DA_130606_E1.h5
DA_130128_E4.h5
DA_130514_E4.h5
DA_130524_E7.h5
DA_130619_E6.h5

Each HDF5 file has the same layout which is described next.

### 1.1 Overall structure

The files contain 5 groups:

- CDD
- DATA
- DYE
- EXPERIMENT
- ILLUMINATION

### 1.2 Details

#### 1.2.1 CCD

The `CCD' group relates to acquisition features with the CCD chip and contains the following **scalar** datasets:

- **GAIN:** the CCD chip gain (from calibration experiments).
- **S_RO:** the read-out standard deviation of the CCD chip (from calibration experiments).
- **P:** the number of pixels in the `Region Of Interest' (ROI); *the read-out variance should be added as many times as there are pixels when one computes the measurement's variance*.
- **P_B:** the number of pixels in the `Background Measurement Region' (BMR).

#### 1.2.2 DYE

The DYE group contains parameters relating to the fluorescent dye, namely the following **scalar** datasets resulting from independent calibration experiments (see the companion manuscript “Analysis of neuronal Ca^2+^ handling properties by combining perforated patch clamp recordings and the added buffer approach” [2] for details):

- **R_min_hat:** the estimated R_min parameter.
- **R_min_se:** the estimated R_min standard error.
- **R_max_hat:** the estimated R_max parameter.
- **R_max_se:** the estimated R_max standard error.
- **K_eff_hat:** the estimated K_eff parameter in (µM).
- **K_eff_se:** the estimated K_eff standard error in (µM).
- **K_d_hat:** the estimated K_d parameter in (µM).
- **K_d_se:** the estimated K_d standard error in (µM).
- **pipette_concentration:** the dye concentration in the pipette in (µM).

#### 1.2.3 ILLUMINATION

The ILLUMINATION group contains parameters describing the illumination used, that is the following **scalar** datasets:

- **T_340:** illumination duration at 340 nm in seconds.
- **T_360:** illumination duration at 360 nm in seconds.
- **T_380:** illumination duration at 380 nm in seconds.

#### 1.2.4 EXPERIMENT

The EXPERIMENT group contains the following datasets:

- AREA: the brain area where the experiment was performed.
- DATE: the experiment date.
- EXPNAME: the experiment name or reference in the lab book.
- LAB: the lab where the experiment was performed.
- OBJECT: the type of neuron recorded.
- OBSERVER: the person who did the experiment.
- ORIGIN: the lab location.
- PROTOCOL: a brief description of the experiment type.
- WEB: URL of the lab where the experiment was performed.

#### 1.2.5 DATA

The DATA group consists of several subgroups which contain the actual calcium imaging data. There is always a ‘load’ group containing the measurements used for monitoring the loading of Fura-2. There are then as many ‘stimX’ groups (*X* = 1,2,…) as stimulations were applied.

Each of these groups contains 3 data sets:

- **ADU:** an array of integers with 7 columns and as many rows as there were measurements. The first column is the time_index column, the second contains the ADU340 measurements (measurements at 340 nm in the ROI), then comes the ADU340B measurements (measurements at 340 nm in the BMR), the ADU360 measurements (at 360 nm in the ROI), the ADU360B measurements (at 360 nm in the BMR), the ADU380 measurements (at 380 nm in the ROI), the ADU380B measurements (at 380 nm in the BMR).
- **TIME_DELTA:** a scalar (see below).
- **TIME_OFFSET:** a scalar (see below).

To get the real time of each measurement, multiply the first column of ADU by TIME_DELTA and add TIME_OFFSET. The other columns of the ADU matrix contain the ‘raw’ readings of the P of P_B pixels from the CCD chip.

## 2 Experimental Design, Materials and Methods

### 2.1 Materials and methods

For methodical details of the brain slices preparation, patch-clamp recordings, and Ca^2+^ imaging, see the companion paper: “*Analysis of neuronal Ca^2+^ handling properties by combining perforated patch clamp recordings and the added buffer approach*” [2]*.* In brief: Electrophysiological data were recorded with an EPC10 patch-clamp amplifier (HEKA, Lambrecht, Germany), which was controlled by the PatchMaster software (version 2.32 for Windows; HEKA). The sampling frequency was 10 kHz. The data were low-pass filtered at 2 kHz with a four-pole Bessel filter. The patch pipettes were fabricated using a vertical pipette puller (PP-830; Narishige, London, UK) from borosilicate glass (0.86 mm inner diameter; 1.5 mm outer diameter; GB150-8P; Science Products, Hofheim, Germany) and had tip resistances between 3 and 5 MΩ. The pipette solution for the whole-cell recordings contained (in mM): 141 K-gluconate, 10 KCl, 10 HEPES, 0.1 EGTA, 2 MgCl_2_, 3 K-ATP, 0.3 Na-ATP and adjusted to pH 7.2 with KOH. Since ATP and GTP can impair the measurement of Ca^2+^ handling properties [4] and to prevent uncontrolled permeabilization of the cell membrane in the perforated patch clamp configuration [5], both were omitted from the pipette solution for recordings in the perorated configuration or Ca^2+^ imaging. In Ca^2+^ imaging experiments, EGTA was substituted by 100 or 200 µM fura-2 (pentapotassium salt, F1200, Molecular Probes, OR, USA). The protocols for the perforated patch recordings were adapted from Fan and Palade [6] and Sarantopoulos [7]. The tips of the patch pipettes were filled with the internal solution. The backfill contained β-escin (∼30–60 µg⋅ml^−1^; E1378; Sigma). The liquid junction potential, which was calculated with the Patcher's Power Tools plug-in from http://www.mpibpc.mpg.de/groups/neher/index.php?page=software for IGOR Pro 6 [Wavemetrics, Lake Oswego, OR, USA], equaled 14.6 mV for normal aCSF and was compensated.

#### 2.1.1 Imaging set up for fluorometric measurements

The fluorometric measurements were performed using an Imago/SensiCam CCD camera with a 640 × 480 chip (Till Photonics, Gräfelfing, Germany) and a Polychromator IV (Till Photonics), both controlled by the program Vision (version 4.0 for Windows, Till Photonics). The filter and mirror settings for the experiments are provided below. Details of the upright microscope are provided in the companion paper [2]. Using 8 × 8 on-chip binning, data were acquired as 80 × 60 frames in arbitrary units (AU) and stored and analyzed as 12-bit grayscale images. For each excitation wavelength, we analyzed the summed fluorescence from a region of interest (ROI) located on the neuron's soma and the summed fluorescence from a background measurement region (BMR), usually larger and located outside of the neuron.

#### 2.1.2 Fluorimetric Ca^2+^ measurements with fura-2

The Ca^2+^ indicator fura-2 was used to determine the intracellular Ca^2+^ concentrations.Fura-2 is a ratiometric indicator suitable to determine absolute Ca^2+^ concentration when calibrated [8,9]. Fura-2 (pentapotassium salt, F1200, Molecular Probes) was loaded into DA SN neurons *via* the patch pipette (100 or 200 µM). It was excited at 340 nm, 360 nm, or 380 nm (410 nm dichroic mirror; DCLP410, Chroma, Rockingham, VT, USA). Emission was detected through a 440 nm long-pass filter (LP440).

#### 2.1.3 Calibration and ‘added buffer approach’

The kinetics of cytosolic Ca^2+^ signals strongly rely on the cell's endogenous and exogenous (added) Ca^2+^ buffers. The amplitude and decay rate of the free intracellular Ca^2+^ change with increasing exogenous buffer concentration: The amplitude of free Ca^2+^ decreases while the time constant increases. With a known added buffer capacity, the time constant of decay can be used to extrapolate the Ca^2+^ signal to conditions, with only endogenous buffers present. The procedure is fully described in the companion manuscript [2].

## Ethics Statement

All animal procedures were conducted in compliance with protocols approved by local government authorities (LANUV NRW, Recklinghausen, Germany) under §4.17.020.

## CRediT authorship contribution statement

**Simon Hess:** Conceptualization, Investigation, Methodology, Visualization, Writing – review & editing. **Christophe Pouzat:** Conceptualization, Methodology, Software, Formal analysis, Data curation, Writing – review & editing. **Peter Kloppenburg:** Conceptualization, Resources, Funding acquisition, Writing – original draft.

## Declaration of Competing Interest

The authors declare that they have no known competing financial interests or personal relationships which have or could be perceived to have influenced the work reported in this article.
